# Progress in Ophthalmology

**Published:** 1902-07-26

**Authors:** 


### PROGRESS IN OPHTHALMOLOGY.

**Asthenopia.**—IN the *Edinburgh Medical Journal*[1] an article appears headed Asthenopia, in which they say: There is no doubt that in the United States the wearing of glasses is much more frequent than in this country. A much larger proportion of the population seem, even to the casual observer, to need such assistance. Presumably, therefore, asthenopia is more common there than here. This relatively greater frequency of asthenopia, says Howe, of Buffalo, U.S., is to be attributed to three causes:—

(1) Carelessness in the use of the eyes, for everyone in America is constantly reading journals, and these journals are larger and worse-printed than those in this country; they are read in trains, "on cars," and at all other equally suitable and unsuitable times. (2) The constant hurry and bustle of ordinary American life is injurious to the nervous system, is conducive to mental and nervous strain, which brings to light imperfections which otherwise might remain innocuous. (3) The American upsets his digestion by eating more meat, and by devouring it more hastily than the Briton; he also consumes too little fluid, certainly less than the average man in other countries. These reasons are all very well and maybe are doubtless true, but the authors of the above journal take exception to his further statement, that "he also wears glasses because the means of testing his refraction, etc., and the opticians are better than in other countries," they say very rightly, it is mistaking cause for effect to say he wears glasses out of admiration for his surgeon's outfit and his optician's mechanical skill. Surely these are developments brought about in answer to the demands of the public.

**Spectacles for Irideremia.**—This is quite a new departure in spectacle making—to provide a proper regulation of the light for eyes that have a congenital absence of the iris. Königshöfer,[2] of Stuttgart, suggests the use of spectacles which, in addition to any necessary lens, are furnished with an apparatus exactly resembling the "iris diaphragm" with which many photographic lenses and microscopes are provided. This, of course, is duplicated, and the controlling rods are connected by a horizontal bar of very light construction, which passes across the lower part of the forehead. Matters are so arranged that when the pupils are medium in size the controlling rods point vertically upwards; should the patient pass into bright light he has merely to push the horizontal bar gently to the left, when both "pupils" at once and equally contract. Should he desire a larger pupil he pushes the bar to the right. Königshöfer says the little apparatus has given great satisfaction to the patient for whom he designed it. The idea seems an excellent one, but we ourselves have not noticed that patients do complain about the light in congenital absence of the iris, and always thought that there must be some compensatory dulling in the sensitiveness of the retina in these cases. One case we can call to mind, where there was not even congenital absence of iris, but where the whole iris was actually torn away as the result of an accident, and even then though we saw the patient many times afterwards there was no complaint made about the worry of too strong light. The rifle experts now sometimes have an iris diaphragm fitted to their orthoptics, and say they find it of great service in the varying lights met with when shooting at their ranges.

**Light and Seating in Schools.**—Zimmerman states his views on this subject in an interesting paper [3]—he says: Since insufficient illumination requires too close an approximation of the book to the eyes, thus fostering the development of myopia, a prime requisite is a certain minimum quantity of illumination for school work. To determine this, the test types of H. Cohn, which have a certain tint, should be read at six metres in sufficient daylight. The main source of illumination should be on the left of the student, so that the hand casts no shadow on the writing. The glass area of the windows should have at least the proportion of one to five of the floor area of the room, and the windows should reach the ceiling. A skylight is very desirable if it can be obtained. The width of the schoolroom—that is the distance of the windows from the opposite wall—must not exceed twice the distance between the desk and the upper window sill. Since the best light is obtained from the sky, the surrounding buildings should be sufficiently low or distant, that the extension of a line drawn from the desk furthest from the window to the edge of the lower window-sill will pass above the roofs. Windows facing south or east give the most intense light, and if the sun is annoying, the writer recommends the use of grey curtains on a pole fastened in the middle of the window, so that the upper or lower half may be darkened separately. Where artificial lighting in schools is necessary, the author recommends a group of incandescent lamps, with lightly ground glass, placed in the centre of the ceiling, with a porcelain reflector above them. Several more may be distributed over the remainder of the ceiling and along the cornices. Another important reform to which attention is called is the correct seating of the students. The bodies of the children should be in such a position that the point of gravity which lies in front of the tenth dorsal vertebra is in a perpendicular line above a base drawn through both seat bones. To maintain this position with comfort, the desk and seat must have certain dimensions and relative positions, conforming to the size of the child, to which requirements the writer draws attention in detail.

[1] Edin. Med. Jour., December 1901. [2] Ibid. [3] Scottish Med. and Surg. Jour., November 1901.

